# The Warmth of Medicine: The Irreplaceability of Doctors in the Era of Artificial Intelligence

**DOI:** 10.2196/97412

**Published:** 2026-06-17

**Authors:** Yu-ying Yang, Jian-min Liu

**Affiliations:** 1 Department of Endocrine and Metabolic Diseases, Rui-jin Hospital, Shanghai Jiao Tong University School of Medicine; Shanghai Institute of Endocrine and Metabolic Diseases; National Clinical Research Center for Metabolic Diseases (Shanghai), 197 Ruijin Er Road, Shanghai, 200025, China, 86 13918221203, 86 21-64333548

**Keywords:** artificial intelligence, doctors, patients, medicine, medical humanities

## Abstract

This article argues that despite the remarkable advances of artificial intelligence (AI) in medicine —including demonstrated capabilities in image recognition, diagnosis, treatment planning, and even empathic communication in controlled settings—the core of medical practice remains irreducibly human. We identify three domains in which AI cannot replace doctors: the holistic, sensory art of clinical observation and intuition; the longitudinal, trust-based doctor-patient relationship built on genuine emotional connection; and the capacity to embrace clinical uncertainty, exercise moral responsibility, and make courageous decisions in the absence of algorithmic guidance. The intended audience includes clinical doctors, medical students, medical educators, and health policy makers navigating the integration of AI into practice. We conclude that preserving “AI-free clinical time” in medical training and safeguarding the humanistic dimensions of care are essential, and technology is to complement rather than diminish the healing arts.

## Introduction

With the rapid advancement of artificial intelligence (AI) technologies, including large language models (LLMs) and multimodal AI, the field of medicine is undergoing unprecedented transformation. AI has made great progress in medical image analysis, nonimage data mining, novel learning paradigms, and human-AI collaborative workflows, and has demonstrated remarkable capabilities in areas such as image recognition, disease prediction, diagnosis, summarizing guidelines, and treatment planning [1,2]. However, no matter how technology progresses, the essence of medicine remains an art that integrates science and humanity, with its core being: “confidence, confidentiality, competence, contract, community responsibility, and commitment” as proposed by David Morrell at 1994 British Medical Association Summit, and then rephrased to “caring, integrity, competence, confidentiality, responsibility, and advocacy” by the conference steering group [3].

It is true that recent clinical studies, including meta-analyses, systematic reviews, and trials, prove AI chatbots and LLM-based AI systems can match or even surpass clinicians in history-taking, following diagnostic pathways, and could deliver more consistent and superior empathetic communication [4-8]. However, these studies were mainly nonclinical, conducted in public online forums and simulated clinical settings, using text-based interactions and standardized patient actors rather than real clinical practice [4-8]. In actual clinical scenarios, the performance of AI in aspects of long-term patient relationships, nonverbal communication rather than synchronous text chat, embodied presence, and professional accountability, especially under clinical conditions of uncertainty and ambiguity, is still not confirmed and is even hard to test. Thus, the notion that AI could replace the central role of doctors is premature and could reflect a “myopic view” [9].

In this paper, we identify three domains in which AI cannot replace doctors: (1) the art of clinical observation and intuition; (2) the trust-based doctor-patient relationship built on genuine emotional connection; and (3) the capacity to embrace clinical uncertainty, exercise moral responsibility, and make courageous decisions in the absence of algorithmic guidance. It is widely accepted that the ultimate goal of medicine is not merely to cure diseases but to care for life—doctors treat the diseases, but more importantly, the patients, the individuals with emotions and dignity. In an era dominated by AI, it is even more crucial for doctors to embody and demonstrate the warmth, wisdom, and value of being human.

## The Art of Medicine: The Significance of Comprehensive History-Taking and Physical Examination

AI can rapidly analyze vast amounts of data, but it cannot fully replace face-to-face communication between doctors and patients. Detailed history-taking and physical examination form the foundation of diagnosis. This process relies not only on objective information but also on the doctors’ decades-honed expertise, experience, intuition, and humanistic care. A patient’s smile or a relaxed conversation can indicate improvement in their condition, while beads of sweat the size of soybeans on the patient’s forehead, cold hands, a trembling voice, and a palpable firm mass allow the physician to gauge the severity and even the malignancy of the disorder. Such comprehensive clinical observation and thinking is an “art” that is difficult for AI to replicate.

We acknowledge that in controlled research settings—particularly text-based evaluations such as Objective Structured Clinical Examination (OSCE)-style assessments—AI models have demonstrated comparable and even better performance than clinicians in history-taking and diagnostic reasoning [4,6]. These findings are valuable and suggest that AI can meaningfully assist in structured clinical encounters. However, the sensory and relational dimensions of bedside medicine—observation of subtle physical signs, tactile information from palpation, and clinical reasoning and judgment abilities formed over years of practice—remain domains in which algorithmic processing cannot yet substitute for embodied human presence.

## The Cornerstone of Doctor-Patient Trust: The Power of Emotional Connection

Doctor-patient trust is “a real human relationship based on love, caring, and sharing” [3]. This trust is built not only on technical competence but also on the empathy and sense of responsibility demonstrated by doctors. When facing illness, patients’ fear and anxiety often extend far beyond the pathology itself. They need love [10]. A comforting word from the doctor, an explanation with patience, or even a reassuring glance can become the motivation for patients to persevere.

AI may be able to provide the “optimal treatment plan,” but it cannot understand a mother’s anguish over her child’s illness or comprehend the desperation of a cancer patient facing death. When a doctor hears a patient with advanced cancer weakly murmur “Why am I still alive?” after a bout of drowsiness, what rises in the doctor’s heart is a complex and heavy mix of emotions—empathy for the patient’s suffering, helplessness at the limitations of medicine, and the struggle with professional ethics. Only through empathy can a doctor help patients and their families understand the disease, accept reality, and participate in decision-making.

This “trust relationship” and empathy are vital components of treatment and the core of medical humanism, which goes far beyond the mechanical delivery of medical information—it requires a keen awareness of the patient’s emotional shifts. When explaining an unfavorable prognosis to patients and their relatives, a doctor should sit facing them, and even hold the patient’s hand, talk *with* rather than talk *to* them. The essence of medicine lies in two-way communication, not one-way information delivery. When a patient hears a diagnosis, their eyes may redden, their voice tremble—subtle body language signaling fear, grief, or helplessness. A doctor can instantly adjust their tone, slow their speech, and say, “I understand this is difficult for you and your family, but we’ll work through it together.” This immediate genuine emotional response is based on the doctor’s own professional characteristics and the empathy developed through the diagnostic and treatment process with patients.

It is noteworthy that current studies challenged the assumption that AI cannot demonstrate empathy. It was found that chatbot responses to patient questions were preferred over physician responses in 78.6% of evaluations and were rated “empathetic or very empathetic” at a rate of 45.1% versus 4.6% for physicians [4], a meta-analysis also demonstrated a mean difference of 0.87 favoring AI over human health care professionals in empathic communication [8]. However, these studies were largely based on single-encounter, text-based interactions evaluated by third-party raters or standardized patients—differs in important ways from the empathy required in ongoing therapeutic relationships. In addition, although AI might also provide some emotionally supportive notes to patients, sometimes its responses are modulated by an inferred assessment of the user’s underlying emotional needs [11]. The longitudinal trust that develops through repeated face-to-face encounters, the shared emotional experience across a disease trajectory, the non-verbal communication of presence during moments of crisis, and the moral commitment to accompany a patient through suffering—these dimensions of the doctor-patient relationship have not yet been systematically evaluated in AI-comparison studies. It is not clear whether patients would maintain the same preference for AI communication when facing a serious diagnosis in person, when making complex trade-off decisions over months of treatment, or at the end of life.

What is more, it cannot be ignored that patients also observe doctors in daily clinical practice to establish his own trust to doctors. Through direct observation and intuition, patients can discern from doctors’ words and demeanor whether the doctors are professional, reliable and steadfast in duty, or arrogant, pretentious and reckless; whether the doctors are ready to take on responsibilities or shift blame when difficulties occur. Such information that patients gain from observing doctors cannot be acquired through AI at present, yet it exerts a vital influence on patients’ decision-making and is closely associated with their physical health and even life safety.

## The Boundaries of Medicine: The “Uncertainty” Beyond AI’s Reach

The value of AI in medicine cannot be judged only by medical efficiency but must also consider the patient’s perspective, including benefits and risks [12]. Interindividual differences— lifestyle habits, social and family background, psychological factors, education level, economic status, and trust in physicians—all influence treatment adherence and outcomes, whereas AI’s “standardized” recommendations may overlook these “non-technical factors.”

Senior doctors learn from both successes and failures. The most valuable aspect of clinical practice lies precisely in those “lessons from failure.” Although extensive quantities of clinical data with negative outcomes are reported in medical corpora and would be used to train AI models, the medical AI systems still face well-documented limitations, such as representational bias [13] and generalizability failure [14]. For example, when using commercial prediction algorithms to manage the health of populations, they exhibited significant racial bias, possibly because the algorithm predicts health care costs rather than illness. This finding suggests the importance of the choice of the label on which the algorithm is trained [13]. Another study revealed that most existing COVID-19 prognostic models show decent discriminative ability but carry high or unclear risk of bias. Their reported performance is overoptimistic and not generalizable to target populations. Such bias arises from model overfitting, improper evaluation (eg, neglect of calibration), and inappropriate management of missing data; thus, “this oversupply of insufficiently validated models is not useful for clinical practice” [14]. Health and disease are complex and holistic; the selection of labels to measure health and diseases is important, while also confusing in constructing a prediction algorithm [13]. Lack of comprehensive understanding of the complexities and uncertainties inherent in medical practice might render their recommendations harboring critical blind spots.

Another concern is that with the widespread application of AI in clinical settings, the new generation of doctors may become increasingly—or even excessively—reliant on algorithmic recommendations, gradually losing their own ability to think independently [2], they may hesitate to explore innovative treatment approaches, and lack the courage to take responsibility for medical decisions.

Furthermore, there are elements in medical practice that AI cannot replace: when technological means are exhausted, a physician’s clinical judgment and perseverance often become decisive factors. When lab or imaging test results are ambiguous, a doctor’s experience and intuition may be life-saving. In moments of public health crises, when confronting unknown lethal diseases without AI support, doctors still charge forward into life-or-death rescues without hesitation. This embodies the sacred and irreplaceable nature of the medical profession.

## Summary: The Symbiosis of Technology and Humanity

AI is a powerful assistant to doctors, but never a replacement. Technological advancement should not come at the cost of medicine’s humanistic care. While technology can optimize workflows and reduce errors, the soul of medicine will always lie in person-to-person compassion. In the era of AI, we must emphasize the core values of doctors more than ever: clinical wisdom, humanistic empathy, responsibility, and courage to make decisions.

The future of medicine is not about machines replacing humans, but about human-machine collaboration—where “AI empowers doctors,” combining AI’s precision with human doctors’ clinical insight to serve patients’ holistic health needs better.

Therefore, in training the next generation of doctors, we must preserve sufficient “AI-free clinical time.” For patients, we should understand that no matter how technology evolves, what they need is not just precise data analysis but also a doctor who listens to their suffering, honors life, offers warmth, and lights their way through darkness—even at life’s final moments. We must safeguard those irreplaceable moments of care—the reassuring smile, the empathetic “I understand your worry,” the intuitive yet life-saving decision. This is the timeless essence of medicine and the fundamental reason human physicians can never be replaced.

Technological progress should never come at the expense of human connection. Science determines how fast we can move forward in medicine; it is humanism that defines how far we can go. Only the warmth of doctors can transform healthcare into a true healing art—one that never loses medicine’s most precious qualities: its humanity and soul.

## Abbreviations

AI: artificial intelligence
LLM: large language model
OSCE: Objective Structured Clinical Examination
